# Acute Aortic Dissection in Monozygotic Twins Presenting Within Days: A Case Report

**DOI:** 10.7759/cureus.99186

**Published:** 2025-12-14

**Authors:** Taysir Al Janabi, Warren Weber, Michael N Vranian, Rahul Kashyap, Avram Flamm

**Affiliations:** 1 Department of Internal Medicine, WellSpan York Hospital, York, USA; 2 Department of Emergency Medicine, WellSpan York Hospital, York, USA; 3 Department of Cardiology, WellSpan York Hospital, York, USA; 4 Department of Research, WellSpan York Hospital, York, USA

**Keywords:** acute aortic syndrome, aortic aneurysms, aortic dissection, monozygotic twin, myocardial infarction

## Abstract

Acute aortic dissection is a serious cardiovascular emergency that needs immediate attention. It is rare for aortic dissection to occur in twins, and there has been no prior report of it happening in a monozygotic twin within three days.

We present the case of a 30-year-old man who presented to the emergency department after an episode of near syncope and was found to have concomitant aortic dissection with inferior myocardial infarction. This case is notable because the patient's twin brother had a repair of Stanford type A aortic dissection just three days prior. The patient underwent an emergent ascending aortic graft and coronary artery bypass graft surgery and recovered after a complex postoperative course.

To the best of our knowledge, this is a rare case report of aortic dissection in a pair of twins that occurred within a three-day span, raising a special interest in a timed genetic mechanism for this occurrence. Additionally, this case highlights the importance of maintaining high suspicion of aortic dissection in the appropriate clinical context and in patients with a relevant family history.

## Introduction

Acute aortic syndromes, including acute aortic dissection (AAD), are a group of uncommon but frequently fatal conditions caused by the disruption of the intimal layer of the aorta, resulting in the separation of the layers of the aortic wall. The incidence rate of AADs in population-based studies was reported to be 4.8 AADs per 100,000 individuals per year [1]. However, those with genetic disorders affecting connective tissue can have AAD at a younger age. Additionally, the relative risk of developing aortic dissection in a sibling of someone with aortic dissection was reported to be 10.9 [2]. The classic symptom of AAD is severe chest pain, tearing in character and radiating to the back. Computed tomography angiography (CTA) is the primary diagnostic modality for classifying AAD into Stanford or DeBakey systems, although transesophageal echocardiography (TEE) and magnetic resonance imaging are reasonable alternatives in certain clinical contexts [3]. AAD is a surgical emergency with high mortality; it has an in-hospital mortality rate of 21.7% for surgically managed type A dissections [4]. Mortality is thought to increase by 1-2% for every hour of delay in management, and if untreated, mortality reaches 50% in the first 48 hours [4]. Myocardial infarction (MI) can share a similar clinical presentation to AAD. Thus, AAD impacting the coronary arteries can add a challenge to the timely diagnosis. We present a case of AAD complicated by inferior MI in a patient whose monozygotic twin also had AAD three days prior.

## Case presentation

A 30-year-old man with a medical history significant for hypertension and morbid obesity (BMI=47) presented to the emergency department (ED) for near syncope, dizziness, and chest tightness. The patient had a family history of a father who had aortic insufficiency status post aortic valve replacement and replacement of the ascending aorta for an aneurysm. 

**Figure 1:**
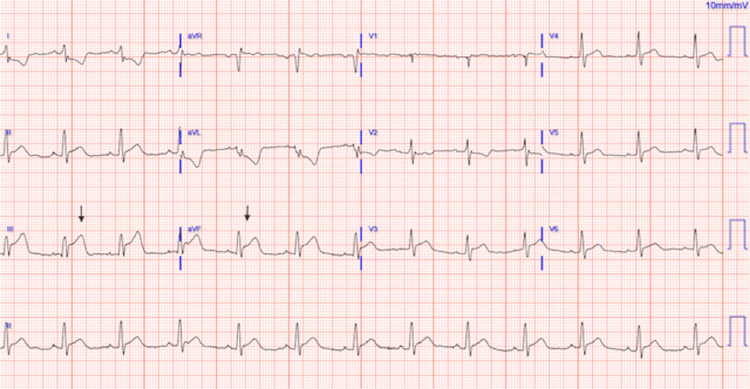
Electrocardiogram upon presentation showing sinus rhythm with acute inferior infarction

The patient was visiting his monozygotic twin brother who was recovering in the cardiothoracic intensive care unit from repair of a type A aortic dissection he had undergone three nights prior. While there, the patient began to feel lightheaded and experienced mid-sternal chest pain, radiating to his back and jaw. The patient was emergently transported to the ED. Physical examination was notable for a pale, diaphoretic young person with equal peripheral pulses. Vitals were significant for blood pressure of 140/90 mmHg. His laboratory workup was significant for leukocytosis of 13.6 K/mcL, creatinine of 1.27 mg/dL, baseline troponin of 17 ng/L, lactate of 9.1 mmol/L, and B-type natriuretic peptide (BNP) of 344 pg/mL (Table 1). A 12-lead electrocardiogram (EKG) was obtained immediately and suggested an inferior ST-segment elevation myocardial infarction (STEMI) (Figure 1). 

**Table 1:** Laboratory findings and their reference values

	Laboratory findings	Reference range
White blood cell (K/mcL)	13.6	4.0-11.0
Creatinine (mg/dL)	1.27	0.70-1.30
Baseline troponin (ng/L)	17	<18
B-type natriuretic peptide (pg/mL)	344	≤100
Lactate (mmol/L)	9.1	0.5-2.0

**Figure 2:**
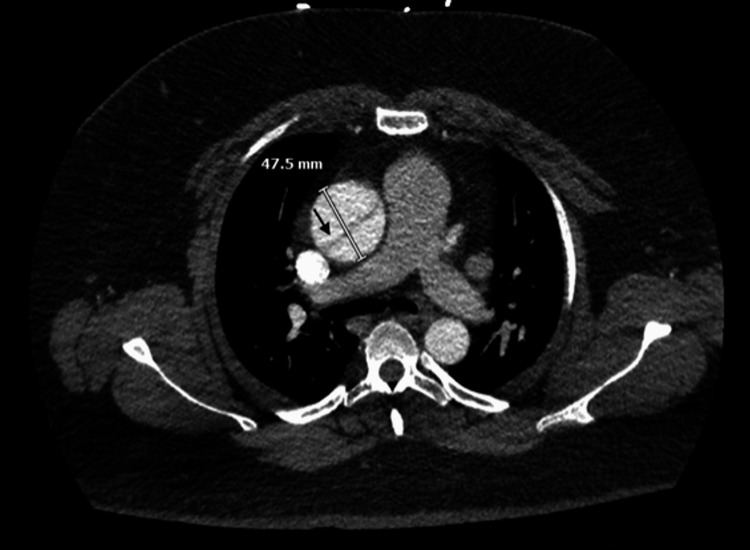
Chest computed tomography angiography showing aortic root dilation and aortic dissection flap as highlighted by the black arrow

**Figure 3:**
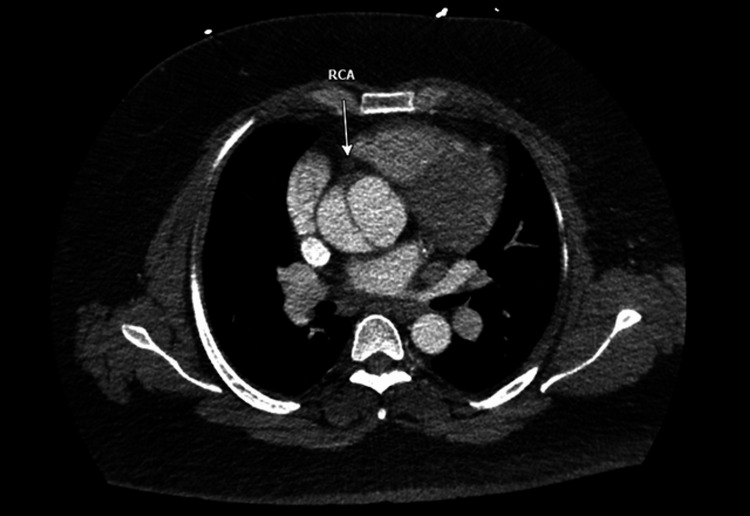
Chest computed tomography angiography showing a dissection flap occluding the ostium of the right coronary artery and lack of opacification of the right coronary artery with contrast as highlighted by the white arrow RCA: right coronary artery

Given the family history and imaging findings, there was a heightened suspicion for AAD. The patient underwent an emergent CTA of the chest, abdomen, and pelvis prior to transport to the catheterization lab. This revealed a type A aortic dissection originating in the aortic root and involving the origin of the right coronary artery. It also involved the origin of the brachiocephalic artery and ended in the proximal descending thoracic aorta (Figures 2-3). 

**Figure 4:**
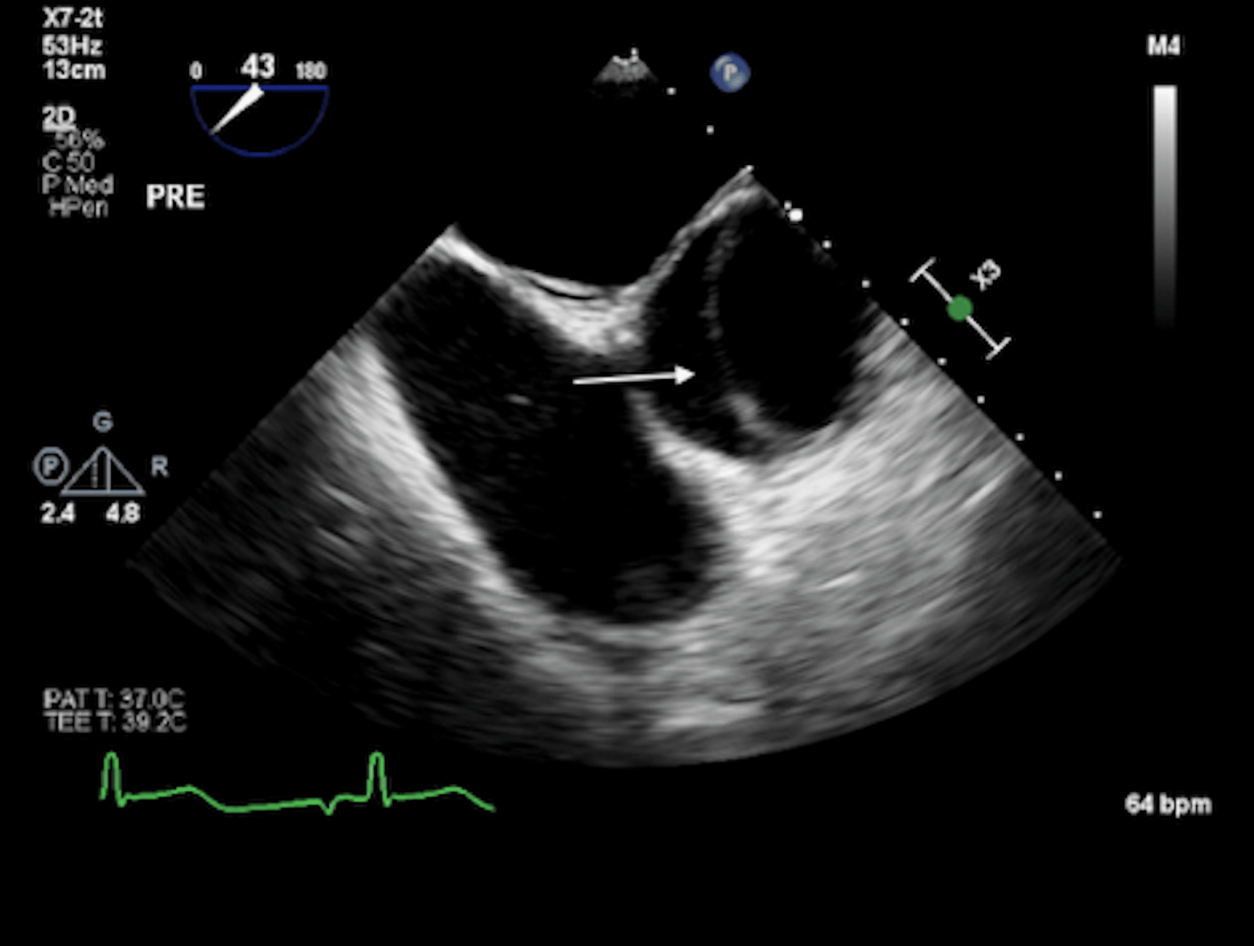
Intraoperative transesophageal echocardiography showing type A aortic dissection with dissection flap as highlighted by the white arrow

Cardiothoracic surgery was consulted, and they recommended starting an esmolol drip, maintaining a systolic blood pressure below 120 mmHg, and activating the operating room (OR) for emergency surgical repair. An intraoperative transesophageal echocardiogram (TEE) showed a dilated aortic root of 48 mm and proximal ascending of 44 mm with a dissection flap in the proximal to distal ascending aorta (Figure 4).

Intraoperatively, the patient was found to have a dissection flap, which involved the origin of the right coronary artery and severe right ventricular (RV) and inferior left ventricular (LV) wall hypokinesia necessitating coronary artery bypass grafting (CABG) of the right coronary artery with a segment of saphenous vein as well as ascending aortic and hemiarch replacement with resuspension of the aortic valve. The postoperative course was complicated by cardiogenic shock, necessitating veno-arterial extracorporeal membrane oxygenation (VA-ECMO) cannulation on postoperative day 1. Additionally, the patient developed acute kidney injury (AKI) requiring temporary continuous renal replacement therapy (CRRT). 

On postoperative day 3, a mediastinal washout with sternal closure was performed in the OR. Intraoperative TEE showed severely reduced systolic function of the LV and RV. By postoperative day 7, the patient tolerated ECMO turndown and was decannulated. Moreover, the postoperative course was complicated by atrial fibrillation requiring cardioversion and heparin-induced thrombocytopenia with a superficial left short saphenous vein thrombus for which he was treated with bivalirudin. The patient was successfully extubated and weaned off from oxygen by discharge. Coumadin was started, and he was transitioned to intermittent dialysis, which was eventually discontinued, and the patient had a normal glomerular filtration rate (GFR) by month 3 after discharge. A genetic test was ordered to evaluate the possibility of a genetic predisposition for AAD, but at the time of this case report, the patient had not completed testing. 

## Discussion

AAD is a catastrophic cardiovascular disease and requires emergent attention. This case is notable regarding the timing of AAD between the patient and his monozygotic twin, which occurred within a three-day time interval. This case report represents the shortest known interval between these two acute aortic syndromes. Redruello et al. reported a monozygotic twin who had two surgeries six months apart for type A aortic dissection and aortic aneurysm of 46 mm, which can potentially develop into aortic dissection [5]. Zhu et al. reported a case of two monozygotic twins who developed an aortic aneurysm in three years [6].

The strong family history, including aneurysms in the patient's father and brother, raises concern for heritable thoracic aortic disease (HTAD). Modern HTAD gene panels now include more than 30 genes known to confer risk for thoracic aortic aneurysm and dissection, encompassing extracellular matrix genes (e.g., FBN1, COL3A1), smooth muscle contractile protein genes (e.g., ACTA2, MYH11, MYLK), and TGF-β signaling genes (e.g., TGFBR1, TGFBR2, SMAD3) [7]. Although genetic testing was ordered for our patient, there is no documentation that it was completed. The near-simultaneous timing of the dissections in the monozygotic twins should raise the question of whether a timed genetic mechanism exists behind the two occurrences. Additional research is needed to explore this question. Familial thoracic aortic aneurysm and dissection have been associated with a few underlying heritable connective tissue disorders. For example, Marfan syndrome is associated with the mutation of the FBN1 gene that codes for fibrillin-1, a protein in the extracellular matrix [8]. 

This case also illustrates the diagnostic challenge of AAD presenting as a STEMI. Our patient arrived with classic inferior ST-segment elevations. Eighty percent of individuals with type A aortic dissection present with chest pain; up to 30% of type A dissections are initially misdiagnosed as another condition, most frequently acute coronary syndrome [9]. Electrocardiographic changes occur in approximately 20% of AAD presentations, and true STEMI occurs in 4-5%, most often involving the inferior wall, as dissection often occurs along the right lateral wall of the ascending aorta, where the shear stress is high [10]. In this case, the treatment team's awareness of the patient's strong family history prompted reconsideration before administering antiplatelet or thrombolytic therapy, thereby preventing potentially catastrophic complications.

This case reinforces the importance of integrating family history, clinical judgment, and targeted imaging when evaluating patients with chest pain and STEMI-like presentations. It also highlights the need for increased awareness of STEMI-mimicking AAD to prevent delays in diagnosis or inadvertent harm from inappropriate therapies. 

## Conclusions

This is a unique case of AAD in a monozygotic twin occurring within three days. This case raises questions about the potential timed genetic mechanisms behind this occurrence and highlights the need for additional research on this topic. Additionally, it is essential to maintain a high level of suspicion for AAD in patients presenting with chest pain. If AAD is suspected in a case of acute coronary syndrome, it is crucial to confirm the diagnosis through appropriate imaging studies as quickly as possible. Failing to do so may lead to the inappropriate use of thrombolytic or anticoagulant medications, which can result in serious complications.
